# An XRE-type regulator in *Streptococcus mutans* plays an important role in *brpA* expression and oxidative stress tolerance response

**DOI:** 10.1128/spectrum.00116-26

**Published:** 2026-07-21

**Authors:** Zezhang T. Wen, Kassapa Ellepola, Lauren C. Guillot, Hemendra P. S. Dhaked, Hui Wu

**Affiliations:** 1 Department of Oral and Craniofacial Biology, School of Dentistry, Louisiana State University Health Sciences Center 12258, New Orleans, Louisiana, USA; 2 Department of Microbiology, Immunology and Parasitology, School of Medicine, Louisiana State University Health Sciences Center 12258, New Orleans, Louisiana, USA; 3 Department of Integrative Biosciences, School of Dentistry, Oregon Health and Science University 6684, Portland, Oregon, USA; Meijo University, Nagoya, Japan

**Keywords:** *S. mutans*, XRE, oxidative stress tolerance response, biofilm formation, *brpA *expression

## Abstract

This study used a functional genomics approach to explore the role of a xenobiotic response element (XRE)-type regulator (SMU.405c) in *Streptococcus mutans* physiology, including the expression of biofilm regulatory protein BrpA. Results showed that deletional mutation of *xre* significantly reduced the ability of the deficient mutant to grow in the presence of methyl viologen, a commonly used oxidative stressor (*P* < 0.001). When challenged in a hydrogen peroxide killing assay, the survival rate of the ∆*xre* mutant was >2-log less than the parent strain after 60 min (*P* < 0.001). Luciferase reporter fusion assays showed that *xre* deficiency had no significant effect on luciferase expression when it was under the control of the intact *brpA* promoter, but the reporter activity increased by >6-fold (*P* < 0.001) when the reporter gene was fused to a *brpA* promoter derivative with deletion of a putative XRE-binding box. Electrophoretic mobility shift assay (EMSA) showed that recombinant XRE interacted with the *brpA* promoter, resulting in an electrophoretic shift of the promoter probes. *In vitro* transcription assay also showed that inclusion of XRE caused transcription to fall off, significantly reducing full-length *brpA* transcripts. RNA-seq analysis revealed that deficiency of XRE led to altered expression of >102 genes by >2-fold (*P* < 0.05), including 28 with increased expression, and 74 with decreased expression. Among the down-regulated were genes for DNA repair and oxidative stress tolerance response. These results suggest that XRE (SMU.405c) in *S. mutans* plays an important role in *brpA* expression and oxidative stress tolerance response.

**IMPORTANCE:**

*Streptococcus mutans*, a keystone pathogen in human dental caries, primarily lives in the highly diverse microbiota on tooth surfaces, where the conditions are often harsh and fluctuate frequently. Locus SMU.405c was annotated to encode a xenobiotic response element (XRE)-like transcriptional regulator, but no information is available concerning the role of this protein in *S. mutans* pathophysiology. This study used a functional genomics approach along with molecular and transcriptomic analysis to characterize a deletional *xre* mutant, and the results showed that *xre* deficiency in *S. mutans* resulted in weakened oxidative stress tolerance response and alterations in transcription of >102 genes, including those known to play an important role in cell envelope biogenesis and stress tolerance response. Reporter fusion assay, electrophoretic mobility shift assay (EMSA), and *in vitro* transcription further demonstrated that the XRE-like regulator encoded by SMU.405c is a repressor of *brpA* expression and plays an important role in oxidative stress tolerance response.

## INTRODUCTION

The oral cavity is featured with fluctuating and often harsh conditions, such as low pH, oxidative stressors, and irregular availability of nutrient sources. Various antimicrobial agents can also be identified from oral care products, such as toothpastes and mouth rinses, such as sodium lauryl sulfate and chlorhexidine, as well as from competing members of the complex microbiota, including acids, hydrogen peroxide, and bacteriocins (antimicrobial peptides) (1). To thrive in the oral cavity, oral bacteria will have to be able to survive and adapt to the different environmental insults; and the bacterial cell envelope plays a vital role during these processes. The envelope serves as both a physical barrier and a molecular sieve, shielding the cells from their environment while providing a platform for cellular components that mediate sensing, signal transduction, and appropriate responses to diverse environmental cues. It is therefore crucial to maintain envelope integrity and ensure proper functions of the cell envelope biogenesis/homeostasis in order for bacterial cells to survive and thrive in certain environmental conditions.

*Streptococcus mutans,* a keystone pathogen of human dental caries, lives almost exclusively in the highly dense, highly diverse plaque microbiota on the tooth surface (2). It is well recognized for its ability to withstand and adapt to diverse environmental insults, a trait central to its pathogenicity. Among the most critical challenges it encounters is low pH, a constant insult brought up largely by its own saccharolytic activity (3). To survive and thrive under such hostile conditions, *S. mutans* employs a multifaceted network of molecular mechanisms that coordinate protein quality control, stress sensing, and transcriptional reprogramming. Molecular chaperones, such as DnaK and GroEL, stabilize nascent or misfolded proteins and facilitate their refolding under acid or oxidative stresses. Complementing this, the caseinolytic protease complexes ClpCP, ClpXP, and ClpEP can degrade irreversibly damaged proteins, thereby preventing toxic aggregation, and maintain protein homeostasis. These proteases are also implicated in regulatory proteolysis, influencing global stress responses and virulence-associated gene expression (4, 5). Besides, two-component signal transduction systems serve as key regulatory nodes that couple extracellular cues to intracellular adaptive programs. For example, CiaHR regulates cell envelope stress responses and contributes to competence development; VicRK modulates cell wall homeostasis, biofilm formation, and virulence factor expression; and LiaSR senses membrane perturbations and orchestrates compensatory gene expression (3, 6, 7).

Biofilm regulatory protein A, BrpA, also plays a major role in stress tolerance responses, including cell envelope stressors and biofilm development, especially when grown in the presence of sucrose (8–10). Compared to *S. mutans* wild type, deficiency of BrpA led to almost complete abolishment of its ability to cause dental caries in a rat caries model (11). Like the other members of the LytR-CpsA-Psr family proteins, BrpA, along with the paralog Psr to a lesser degree, functions as a ligase, promoting the attachment of rhamnose-containing glucose polymers to the peptidoglycan, significantly influencing bacterial pathophysiology in *S. mutans* (12). Our recent studies have shown that the expression of *brpA* is regulated in response to environmental cues, and multiple *cis-* and *trans-*acting factors play an important role in the regulation of *brpA* expression, including an Fnr-box, a major repressor (Fig. S1) (13).

The xenobiotic response element (XRE) family transcriptional regulators are some of the most common transcriptional factors in bacteria, regulating the expression of a wide range of genes, including those for virulence factors, key enzymes of metabolic pathways, antibiotic synthesis and resistance, cell envelope biogenesis, stress tolerance response, and biofilm formation (14–18). *S. mutans* possesses at least seven XRE-like proteins (19), although currently there is little information available concerning the exact roles these regulators play in *S. mutans* pathophysiology. In this study, a gene (locus SMU.405c or SMU_RS01970 in NCBI annotation) encoding an XRE-like protein is identified proximately upstream of *brpA* (SMU.410) (Fig. S2). A functional genomics approach was used to characterize the roles of the SMU.405c-encoded protein, herein termed XRE, in *S. mutans* physiology, including *brpA* expression. The results showed that XRE is a negative regulator of BrpA expression and plays a role in *S. mutans’* stress tolerance response and biofilm formation.

## MATERIALS AND METHODS

### Bacterial strains and growth conditions

All bacterial strains used and created in this study are listed in Table 1. Unless indicated specifically, *S. mutans* UA159 and its derivatives were maintained and grown in Brain Heart Infusion (BHI; Becton, Dickinson and Company, Sparks, MD, USA) in an aerobic chamber at 37°C plus 5% CO₂. Antibiotics—erythromycin (Sigma, St. Louis, MO, USA) (10 μg mL^−1^), spectinomycin (Sigma) (1 mg mL^−1^), and/or kanamycin (Sigma) (1 mg mL^−1^)—were added to the growth medium whenever necessary. *Escherichia coli* strains were grown in an aerobic incubator at 37°C in LB broth with or without shaking; and when needed, erythromycin (Sigma, St. Louis, MO, USA) (300 μg mL^−1^), spectinomycin (Sigma) (100 μg mL^−1^), ampicillin (100 μg mL^−1^), and/or kanamycin (Sigma) (40 μg mL^−1^) were added to the growth medium. For agar medium plates, Bacto agar (Becton, Dickinson and Company, Sparks, MD, USA) was added at 1.5% wt/vol.

**TABLE 1:** Bacterial strains and plasmids used in this study^*a*^

Strains/plasmid	Major characteristics	References
*S. mutans* UA159	Wild type	ATCC700610
*S. mutans* TW487	UA159/∆*xre* (SMU.405c), kan^r^	This study
*S. mutans* TW487e	UA159/∆*xre* (SMU.405c), erm^r^	This study
*S. mutans* TW487c	UA159/∆*xre/gtfA::xre*, erm^r^, kan^r^	This study
*E. coli* DH10B	Cloning host, *mcrA, mcrBC, mrr,* and *hsd*	Invitrogen, Inc.
*E. coli* M15	Expression host	Qiagen, Inc.
pQE30	Expression vector	Qiagen, Inc.
pFW11-*luc*	Integration vector containing a promoterless luciferase gene, Spc^r^	20
pBGK3	Integration vector	13
pFW11::Pldh::luc	Luciferase reporter fused with the lactate dehydrogenase gene promoter *Pldh*, Spc^r^	13
SP3	pFW11::P*brpA::luc,* luciferase gene fused to the short intact *brpA* promoter	13
dF	pFW11::P*brpA*∆Fnr-box*::luc,* luciferase gene fused to the short *brpA* promoter with Fnr-box and its 3′ deleted	13
dX	pFW11::P*brpA*∆XRE-box*::luc,* luciferase gene fused to the short *brpA* promoter with XRE-box deleted	This study

^*a*^ Kan^r^, Erm^r^, and Spc^r^ for kanamycin, erythromycin, and spectinomycin resistance, respectively.

### Functional genomic analysis

Standard recombinant DNA techniques were employed unless they are stated otherwise. All related agents, including restriction and modifying enzymes, were purchased from New England Biolabs (Beverly, MA, USA) and/or Invitrogen (Thermo Fisher Scientific, Life Technologies, NY, USA) and used following the manufacturers’ guidelines. Allelic exchange ∆*xre* mutants were constructed using the PCR-ligation-mutation strategy with gene-specific primers (Table S1); and Sanger sequencing was used to verify sequence accuracy (21). For the ∆*xre* mutant complementation, the wild-type *xre* gene plus its cognate promoter was PCR amplified and integrated via double crossover recombination at the *gtfA* site via integration vector pBGK3 (12).

To assess the effect of *xre* deletion on *brpA* expression*,* the resulting mutant was transformed with the promoter-luciferase reporter fusions of interest (13), and the impact of XRE-deficiency on *brpA* expression was analyzed by luciferase assays (13, 20). *S. mutans* carrying the luciferase reporter fused with the constitutive promoter of the lactate dehydrogenase gene *ldh* was also used as a reference (Table 1).

To characterize the effect of *xre* deficiency on *S. mutans* growth kinetics, Bioscreen C was used with bacterial strains grown in regular BHI, BHI adjusted to pH 6.0, BHI with inclusion of methyl viologen at 25 and 12.5 mM, and BHI with sodium nitroprusside at 2 mM (22). For its impact on *S. mutans* stress tolerance responses, acid killing and hydrogen peroxide challenge assays were carried out as previously described (23). Effects on *S. mutans* biofilm formation were analyzed using 96-well plates via crystal violet staining and on hydroxyapatite (HA) disc via confocal laser scanning microscopy with bacterial strains grown in biofilm medium with glucose (20 mM), sucrose (20 mM), and glucose plus sucrose (18, 2 mM) as described previously (21, 24).

### Recombinant protein expression and purification

For expression and purification of recombinant XRE protein (rXRE), the *xre-*coding region was amplified by PCR using high-fidelity DNA polymerase Q5 (NEB Biolabs) (Table S1) and cloned in pQE30 (Qiagen, Inc.). Following Sanger sequencing to confirm the accuracy of the cloned DNA, the resulting construct was transformed into *E. coli* M15, and the His-tagged recombinant protein was expressed by the addition of isopropyl β-D-1-thiogalactopyranoside at 0.5 mM, and then purified using nickel resins (Fisher Scientific) under native conditions, similarly as described previously (25). The purified protein was then reconstituted in phosphate-buffered saline, pH 7.6, plus 20% glycerol using Amicon-10 Centrifugal Filters (10 kDa MWCO, Millipore).

### *In vitro* transcription assay

*In vitro* transcription assays were carried out using *S. mutans* RNA polymerase and recombinant sigma factor A (SigA) with and without inclusion of rXRE, similarly as described by Ellepola et al. (26). The resulting transcripts were analyzed using 1.4% denaturing agarose gel and visualized by SYBR Gold staining (Invitrogen, CA).

### EMSA assay

Electrophoretic mobility shift assay (EMSA) was carried out similarly as described previously; and DNA mobility shift was visualized via LightShift Chemiluminescent EMSA Kit (Pierce, Rockford, IL) (25, 26).

### RNA-seq and qRT-PCR analysis

For RNA-seq, mid-exponential phase cultures with OD_600nm_ ~0.4 were treated with RNAProtect (Qiagen Inc., CA), and total RNAs were extracted using a ZymoBIOMICS Quick-bacteria and fungi RNA Miniprep Kit (Zymo Research) by following the supplier’s guidelines (8, 27). The RNA samples were depleted of ribosomal RNA, and libraries were generated using Illumina’s Stranded Total RNA Prep Ligation with the Ribo-Zero Plus kit. Sequencing was done at the SeqCenter (Pittsburgh, PA) on an Illumina NovaSeq X Plus platform, producing paired-end 150 bp reads; and demultiplexing, quality control, and adapter trimming were performed with bcl-convert (v4.2.4). Processed/cleaned reads were mapped to the *S. mutans* UA159 genome (GCF_000007465.2) with HISAT2 (v2.2.1) using default parameters + “very sensitive” (28). Differential expression analysis was performed using edgeR’s glmQLFTest with those with a log2(fold change) > 1 and a *P* < 0.05. More details on RNA-sequencing and post-seq analysis can be found in the Supplemental Materials. Selected genes were further analyzed using RealTime-qPCR by following our established protocols with 16S rRNA as the control (8, 27). Gene expression data is deposited in the NCBI Gene Expression Omnibus (GEO) database (https://www.ncbi.nlm.nih.gov/geo/) under GEO Series Accession number GSE314968.

### Statistical analysis

To ensure rigor, all experiments were repeated with proper controls and at least three independent times. Unless stated otherwise specifically, the results were analyzed using Student’s *t*-test for differences between different groups/conditions, and a difference of *P* < 0.05 was considered statistically significant.

## RESULTS

### XRE-deficiency affects oxidative stress tolerance of the deficient mutant

**Fig 1:**
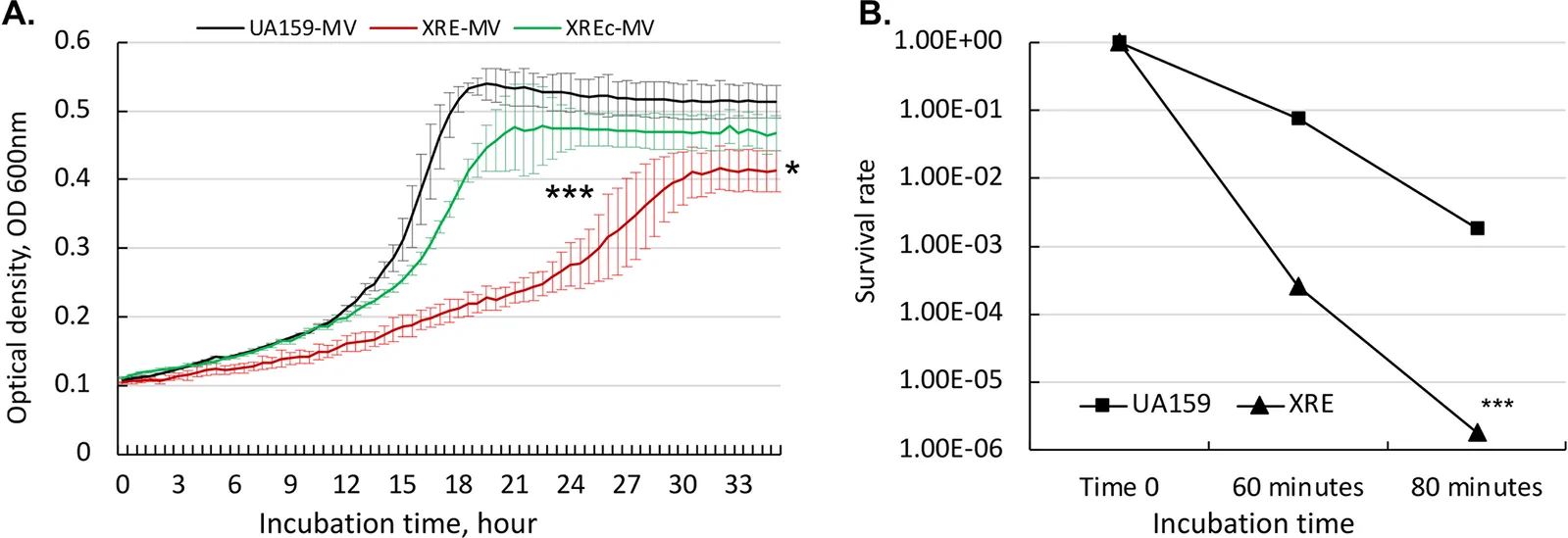
Growth characterization (**A**) and hydrogen peroxide challenge assay (**B**). (**A**) *S. mutans* UA159, its ∆*xre* mutant (XRE), and complement strain (XREc) were grown in BHI broth with inclusion of methyl viologen (MV, 25 mM) in a Bioscreen C. Data are representative of three independent experiments. (**B**) *S. mutans* UA159 and its ∆*xre* mutant were incubated in 0.1 M glycine buffer, pH 7.0, containing 58 mM hydrogen peroxide for periods of 60 and 80 min; and the results in survival rate showed that the ∆*xre* deficiency resulted in a significant reduction in survival against the oxidative stressor, compared to its parent strain, UA159. *, *** indicating *P* < 0.05 and 0.001, respectively.

Locus SMU.405c (SMU_366, SMU_RS01970) is annotated to encode a protein of 136 amino acids with homology to the XRE family of transcriptional regulators (19). A deletional mutant was constructed using a non-polar erythromycin-resistance element to replace nucleotides 47 to 214 of its coding region. When compared to the parent strain, UA159, the *xre* deletional mutant, TW487, had a tendency to form aggregates when growing in BHI broth. Otherwise, it did not show any major differences in growth rate, doubling time, and colony morphology on BHI medium in comparison to the parent strain. When analyzed using a Bioscreen C, the ∆*xre* mutant displayed similar doubling time and optical density overnight as its parent strain, UA159, when they were growing in regular BHI broth (Fig. S3A). However, when they were growing in BHI with inclusion of methyl viologen at 25 mM, the ∆*xre* mutant displayed a major reduction in growth rate with a doubling time of 870 (±67) min vs 240 (±35) min for UA159 (*P* < 0.001), and a reduced optical density overnight with an average of 0.41 (±0.04) vs 0.54 (±0.15) for the parent strain (*P* < 0.05) (Fig. 1A). Similar effects, but to a lesser degree, were also observed when grown in the presence of 12.5 mM methyl viologen (data not shown). When incubated in glycine buffer, 0.1 M, pH 7.0, with 58 mM hydrogen peroxide in a killing assay, the survival rate of the ∆*xre* mutant was reduced by >2-logs in 60 min and >3-logs after 80 min (*P* < 0.001), compared to the wild type (Fig. 1B). As expected, the complement strain, TW487c, with the wild-type *xre* plus its cognate promoter in a single copy, restored the phenotypes in both growth rate and overnight culture density to a level similar to the wild type, UA159 (Fig. 1A). No major differences were measured when they were growing in BHI adjusted to pH 6.0 or BHI with inclusion of sodium nitroprusside (Fig. S3B and D).

### XRE deficiency displayed moderate effects on biofilm formation by the deficient mutant

**Fig 2:**
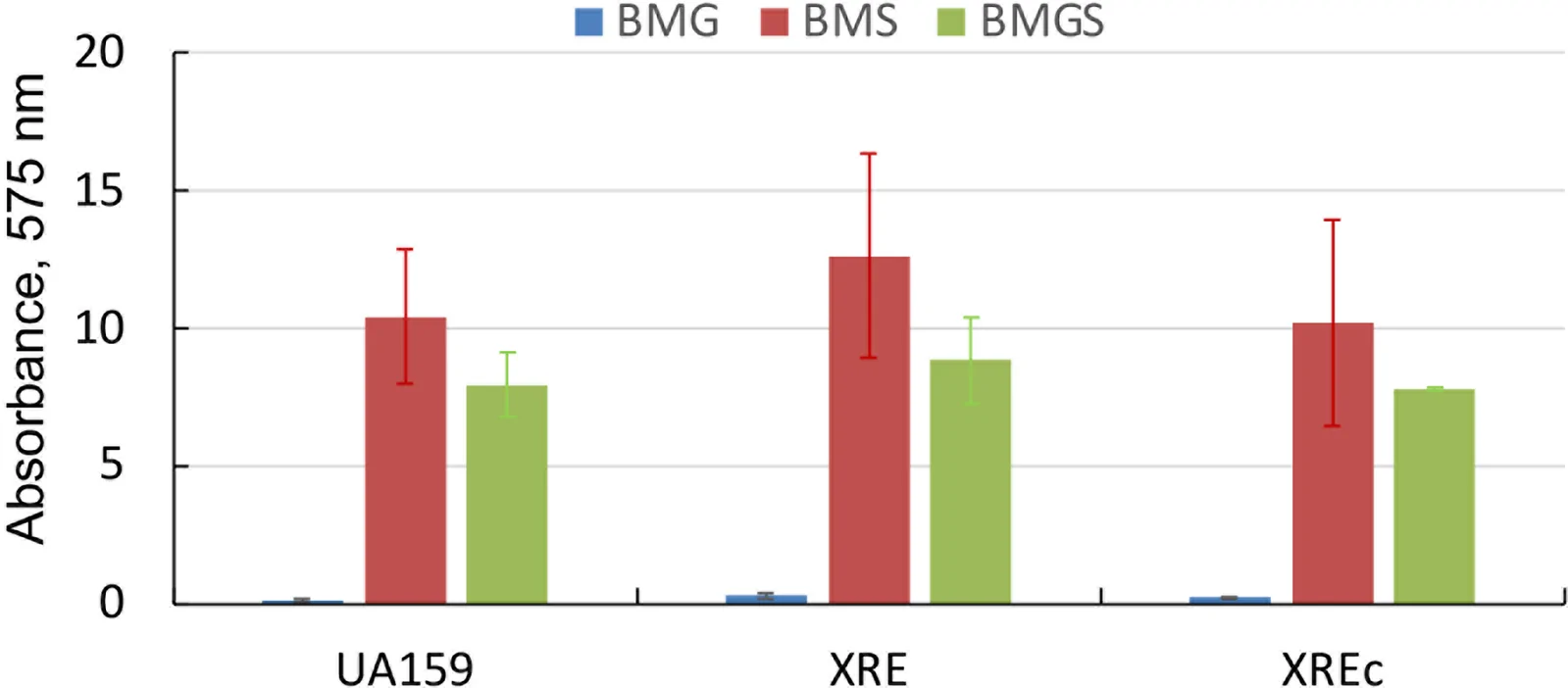
Biofilm formation by the ∆*xre* mutant. *S. mutans* UA159 (UA159), the ∆*xre* mutant (XRE), and its complement strain (XREc) were grown in biofilm medium with glucose (BMG), sucrose (BMS), and glucose plus sucrose (BMGS) on 96-well plates and stained with crystal violet. The ∆*xre* mutant was shown to form more biofilm, but such enhancement was not statistically significant when analyzed via paired Student's *t*-test, *P* > 0.05.

**Fig 3:**
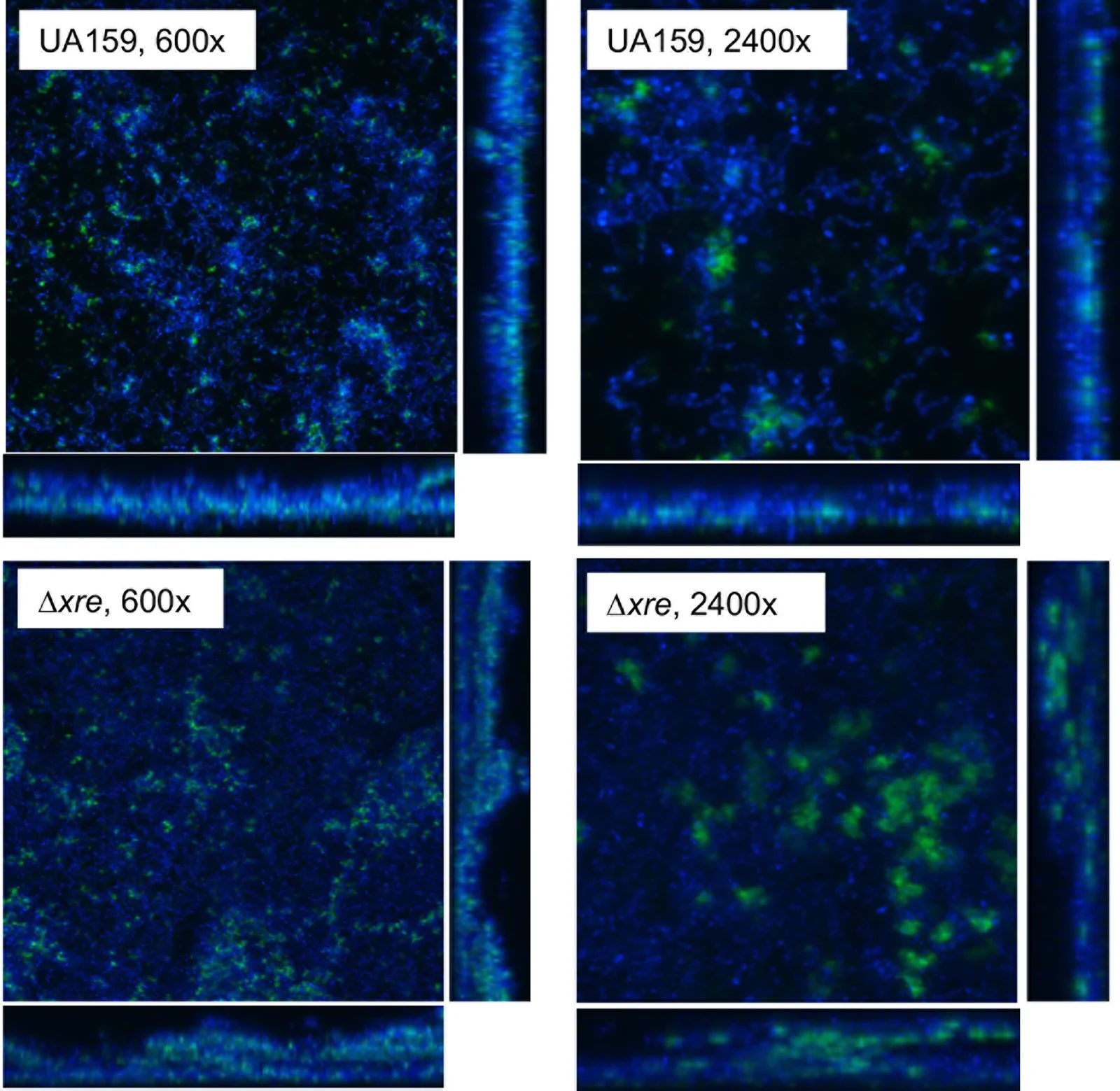
Confocal microscopic analysis of ∆*xre* mutant biofilms. *S. mutans* UA159 and its ∆*xre* mutant were grown in BM medium with glucose plus sucrose overnight; and biofilms on hydroxyapatite discs were stained with Alexa Fluor 488-conjugated ConA and Hoechst prior to confocal analysis, which confer the glucans with green fluorescence and cellular DNA with blue fluorescence, respectively. Images presented are representatives of compressed xyz, xz, and yz from three different sets of experiments.

When analyzed using a 96-well plate model and crystal violet staining, the ∆*xre* mutant was shown to form slightly more biofilms on the polystyrene surface, especially when it was growing in the presence of sucrose, although such differences were not statistically significant when compared to its parent strain under the conditions studied (*P* > 0.05) (Fig. 2). When grown on hydroxyapatite discs and analyzed under a confocal laser scanning microscope, the wild-type biofilms appeared to grow and accumulate evenly on the surface, while the ∆*xre* mutant existed mostly in larger aggregates of intercellular clusters (Fig. 3), similar to the results of the 96-well plate model. When Alexa Fluor 488-conjugated ConA (Ex/Em 495/519 nm, ThermoFisher Scientific) and Hoechst 33342 (Ex/Em 350/461 nm, ThermoFisher Scientific) staining was used, which confers extracellular glucans with green fluorescence and the microbial DNA with blue fluorescence, respectively (24, 29), the ∆*xre* mutant biofilms were found to contain somewhat more green fluorescent glucans than the UA159 biofilms, although again there were no significant differences statistically when the optical images were further analyzed quantitatively using ImageJ-Fiji under the conditions studied (Fig. 3).

### XRE plays a role in regulation of *brpA* expression

**Fig 4:**
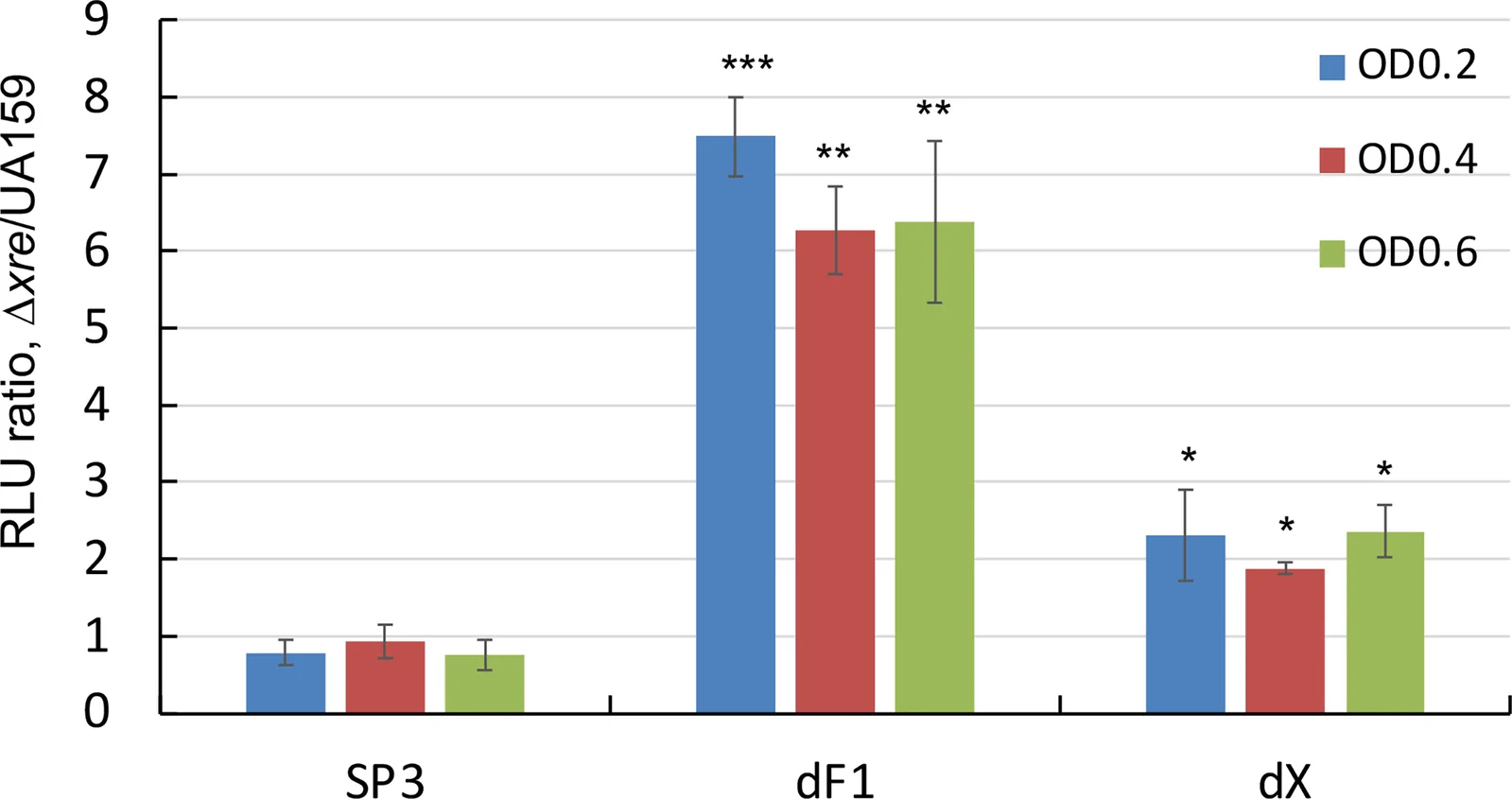
Reporter fusion assays of the ∆*xre* mutant. *S. mutans* UA159 and its ∆*xre* mutant carrying the reporter fusions were grown in BHI until early- (OD_600nm_ of 0.2), mid- (OD ~0.4), and late- (OD ~0.6) exponential phase. Data presented here are expressed as ratios of the luciferase reporter activities of the ∆*xre* mutant over UA159 under the direction of intact short promoter (SP3), SP3 with Fnr-box and its 3′ deleted (dF1), and XRE-box mutation (dX) with *, **, ***, indicating *P* < 0.05, 0.01, and 0.001, respectively, compared to SP3 at the same OD via Student's *t*-test.

In consideration that the locus SMU.405c localizes closely upstream of *brpA,* the effect of XRE-deficiency on *brpA* expression was first analyzed using luciferase reporter fusion assays. As compared to the wild-type strain, when the luciferase reporter was expressed under the direction of the intact *brpA* promoter, SP3 (Fig. S1), the luciferase reporter activity in the ∆*xre* mutant did not show any significant differences (*P* > 0.05) (Fig. 4) (13). However, when the luciferase reporter gene was directed under promoter derivative dF1 that has the Fnr-box, a major repressor, and its 3′ end deleted, the reporter activity in the ∆*xre* mutant was increased by >6-fold (*P* < 0.001), compared to the wild-type UA159 (Fig. 4), especially during the early-exponential phase (OD_600nm_ ~0.2), when *brpA* expression is at its maximum.

A closer examination of the *brpA* promoter identified a region, AttTCTTGATGTT, located at nucleotides (nt) −38 to −50 relative to the transcription initiation site ATG (Fig. S1), with high similarity to the XRE-binding sequence ACCN(0,7)GTT (17) (lower case indicating mismatches to the XRE-box consensus). It is partly overlapping the Fnr-box, located nt −30 to −43 (13). An internal deletion of TTGATGTT of the XRE-box was conducted; and the impact of such deletion on *brpA* expression in the wild type and the ∆*xre* mutant was analyzed by the luciferase reporter assay. As expected, similar results as the Fnr-box and its 3′ end deletion, but to a lesser degree, were also observed when the luciferase reporter was fused with the *brpA* promoter with internal deletion of the XRE-box (dX).

**Fig 5:**
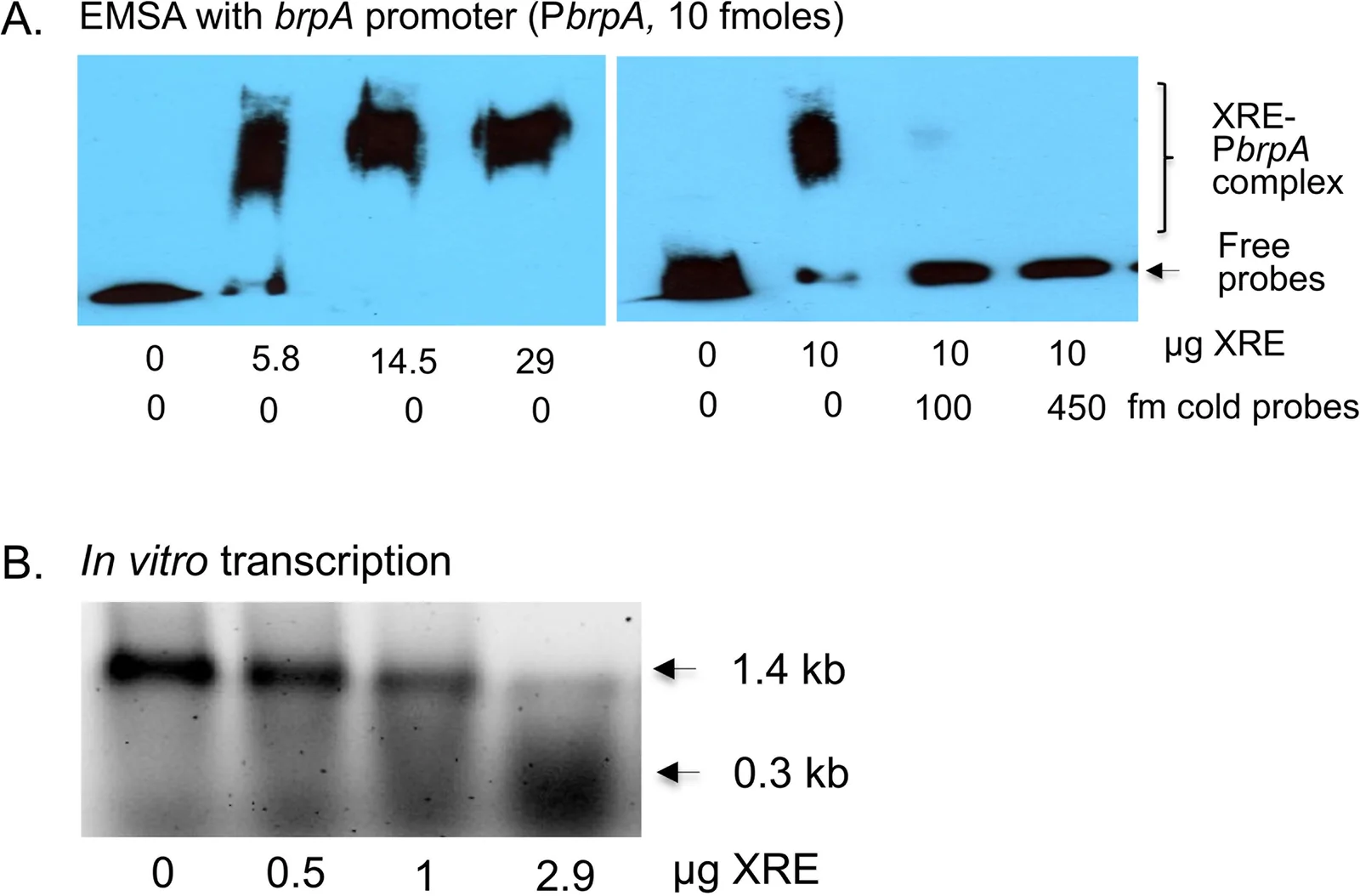
EMSA (**A**) and IVT analysis (**B**). (**A**) EMSA showed that inclusion of recombinant XRE resulted in mobility shift of biotinylated *brpA* promoter probes, while prior incubation of XRE with cold probes led to abolishment of such electrophoretic shift. (**B**) When analyzed using IVT with a *brpA-*coding region plus its intact short promoter as the template, inclusion of XRE in the reaction was shown to result in a major reduction of *brpA* transcripts, indicating rXRE as a repressor. Data presented are representative of more than three independent experiments.

XRE is predicted to possess a helix-turn-helix DNA-binding domain in the N-terminus (Fig. S4a). To further assess if XRE interacts with the *brpA* promoter, a rXRE was expressed and purified by affinity chromatography (Fig. S4b), and EMSAs were carried out with and without inclusion of rXRE. As shown in Fig. 5A, inclusion of rXRE in EMSAs led to electrophoretic shift of the biotinylated promoter probe. Consistently, in a cold-competition assay, prior incubation of rXRE with un-biotinylated probes resulted in no electrophoretic shift of the biotinylated probes. These results suggest that XRE is capable of interacting with the *brpA* promoter.

*In vitro* transcription assay was also used to examine if and how rXRE influences the transcription of *brpA* with the *brpA-*coding region plus its intact promoter as the template. As shown in Fig. 5B, inclusion of rXRE in the assay mixture led to a reduced level of *brpA* transcription, and such effects were shown to be concentration-dependent. With increasing amounts of rXRE in the reaction mixture, the amount of *brpA* transcripts of ~1.4 kb was reduced further, while a small transcript of ~0.3 kb, a likely result of transcription fall-off, was increased. These results further suggest that XRE is a negative regulator of *brpA* expression under the conditions studied, consistent with the results of luciferase reporter fusion assays.

### XRE-deficient mutant has an altered transcriptome

To further investigate what other genes the XRE (SMU.405c)-like regulator may regulate under the conditions studied, RNA-seq analysis was conducted with total RNA extracted from cultures grown until mid-exponential phase (OD_600nm_ ~0.4). As illustrated by the Heatmap (Fig. S5), some major differences existed between the transcriptomic profiles of the ∆*xre* mutant and its parent strain, UA159. Relative to the wild-type UA159, deficiency of XRE led to altered expression of >102 genes by >2-fold (*P* < 0.05), including 28 genes that were increased in their expression and 74 genes that decreased expression (Table 2). Among the up-regulated genes are those for glucosyltransferase B (*gtfB*) and glucan-binding protein C (*gbpC*), two major factors known to play central roles in *S. mutans* adherence and accumulation of multi-cellular clusters during biofilm formation. RealTime-qPCR was used to further evaluate the differential transcription of *gbpC* and *gtfB,* and the results showed that *gbpC* was measured with 8.12E7 (±9.51e6) copies for the mutant vs 1.46E7 (±2.21E6) copies for the parent strain (*P* < 0.05); and *gtfB* was averaging 1.63E9 (±1.08E8) copies for ∆*xre* mutant vs 7.4E8 (±7.2E7) copies for the wild type (*P* < 0.05). The up-regulated genes also include *uvrC* for excinuclease ABC subunit C and *mubX* (SMU_RSO6200 and SUM_RSO6240) for mutanobactin A system ABC transporter ATP-binding subunits MubX.

**TABLE 2:** Up-regulated and down-regulated genes in response to XRE-deficiency

Up-regulated genes in response to XRE-deficiency^*a*^				
Locus tag	Gene	Description/potential functions	Fold change (X/W)	*P* value
SMU_RS00485		TIGR01440 family protein	2.18187637	0.000378533
SMU_RS01370		PTS sugar transporter subunit IIA	2.305907235	0.008056966
SMU_RS01455		HXXEE domain-containing protein	2.115196612	0.000307864
SMU_RS02200		Hypothetical protein	2.095911749	2.37689E-05
SMU_RS02315	rsmB	16S rRNA [cytosine(967)-C(5)]-methyltransferase RsmB	2.72126442	0.024165659
SMU_RS02550		Chorismate mutase	2.409587423	0.001277449
SMU_RS02610		Rhodanese-like domain-containing protein	2.017629528	1.17528E-05
SMU_RS02835		Dihydroorotate oxidase	2.878969038	1.45868E-06
SMU_RS03000		VIT1/CCC1 transporter family protein	3.032515255	1.05963E-06
SMU_RS03570		IS3-like element ISSmu1 family transposase	10.06580334	0.002325592
SMU_RS04500	licT	BglG family transcription antiterminator LicT	2.220306806	1.77209E-05
SMU_RS04620	gtfB	Glucosyltransferase GtfB	2.011202394	0.00152629
SMU_RS05505		DUF3862 domain-containing protein	2.001990631	0.003766141
SMU_RS05730	uvrC	Excinuclease ABC subunit UvrC	2.433457611	0.0283103
SMU_RS06200	mubX	Mutanobactin A system ABC transporter ATP-binding subunit MubX	2.883485584	0.0150399
SMU_RS06210		IS3 family transposase	2.007815557	0.000246381
SMU_RS06240	mubX	Mutanobactin A system ABC transporter ATP-binding subunit MubX	2.80311869	0.017634663
SMU_RS06360	gbpC	Glucan-binding protein GbpC	2.546611469	0.000730688
SMU_RS06410		IS3-like element ISSmu1 family transposase	3.413400128	0.000727922
SMU_RS06985	glgC	Glucose-1-phosphate adenylyltransferase	3.796304423	0.002640708
SMU_RS07045		Hypothetical protein	2.472405728	0.000722005
SMU_RS07370		CvfB family protein	2.069092577	3.91325E-05
SMU_RS07420		NUDIX hydrolase	2.231804384	1.65443E-06
SMU_RS09040		DUF1033 family protein	2.337256825	0.00060638
SMU_RS09905		Hypothetical protein	2.461005732	0.029080243
SMU_RS09940		Hypothetical protein	2.677015834	0.03783558
SMU_RS10050		Type II toxin-antitoxin system PemK/MazF family toxin	2.943998454	0.008856824
SMU_RS10070		Hypothetical protein	2.931954086	0.001873147
SMU_RS10140		Hypothetical protein	2.447910321	0.001345002

Down-regulated genes in response to XRE-deficiency^*b*^				
Locus tag	Gene	Description/potential functions	Fold change (W/X)	*P* value
SMU_RS00420		PFL family protein	2.594520109	0.03965201
SMU_RS00525		PTS system mannose/fructose/N-acetylgalactosamine-transporter subunit IIB	2.299711178	0.000870294
SMU_RS00700		Malolactic enzyme	2.737644876	0.000494408
SMU_RS00705		AEC family transporter	2.809028683	0.00243998
SMU_RS00710		Oxalate decarboxylase family bicupin	2.571857978	0.002519909
SMU_RS00715		Dihydrolipoyl dehydrogenase family protein	2.42551979	0.003433783
SMU_RS00720		YoaK family protein	2.384126058	0.000967742
SMU_RS01010		Site-specific integrase	3.222892914	0.002806723
SMU_RS01015		DUF3173 domain-containing protein	19.67976046	0.003611071
SMU_RS01020		ASCH/PUA domain-containing protein	4.772324059	0.001410147
SMU_RS01025		Hypothetical protein	4.998339714	0.001333747
SMU_RS01030		Phage tail tip lysozyme	5.330614685	0.000577515
SMU_RS01035		Hypothetical protein	5.060282976	0.001156324
SMU_RS01040		ATP-binding protein	4.445854585	0.002614555
SMU_RS01045		Conjugal transfer protein	6.120404526	0.000599801
SMU_RS01050		TcpD family membrane protein	3.232360676	0.009361105
SMU_RS01055		Conjugal transfer protein	5.252013151	0.003364161
SMU_RS01060		Hypothetical protein	4.675110908	0.006754871
SMU_RS01065		Hypothetical protein	4.461779943	0.001831096
SMU_RS01075		Replication initiation factor domain-containing protein	5.350709081	0.00058084
SMU_RS01080	SMC, SMU.1513	FtsK/SpoIIIE domain-containing protein	5.059024846	0.000182728
SMU_RS01085		DUF961 family protein	5.665584432	0.000178125
SMU_RS01090		Hypothetical protein	4.410091249	0.000622687
SMU_RS01095		Hypothetical protein	2.624113605	0.003802991
SMU_RS01100		Hypothetical protein	2.37781068	0.044705005
SMU_RS01105		Hypothetical protein	2.929610948	0.000866237
SMU_RS01110		Hypothetical protein	2.673867204	0.005943656
SMU_RS01115		Hypothetical protein	2.187405675	0.019343936
SMU_RS01965		Hypothetical protein	2.33324581	0.001099
SMU_RS01970	xre1, SMU.405	Helix-turn-helix domain-containing, XRE family transcription regulator	59.66806585	1.64598E-12
SMU_RS02390		DEAD/DEAH box helicase	8.436572671	5.99438E-07
SMU_RS02895		SH3 domain-containing protein	2.56817306	0.001371075
SMU_RS02950		Helix-hairpin-helix domain-containing protein	5.269988035	1.09253E-06
SMU_RS02955		DNA internalization-related competence protein ComEC/Rec2	7.380013003	0.000315126
SMU_RS03145	argJ	Bifunctional ornithine acetyltransferase/N-acetylglutamate synthase	3.060577489	0.017142382
SMU_RS03875		GBS Bsp-like repeat-containing protein	2.393360776	7.54568E-05
SMU_RS04035		DeoR/GlpR family DNA-binding transcription regulator	3.434558268	0.012697743
SMU_RS04060		IS3 family transposase	7.811808304	0.003885808
SMU_RS04510		Prolyl oligopeptidase family serine peptidase	16.8448704	0.003590605
SMU_RS04605	dprA	DNA-processing protein DprA	5.418008728	1.19162E-05
SMU_RS05020		FAD:protein FMN transferase	2.024844006	0.004020736
SMU_RS05455		PTS mannitol transporter subunit IICB	4.577943888	0.00335986
SMU_RS05825	hisF	Imidazole glycerol phosphate synthase subunit HisF	4.151412657	0.037024144
SMU_RS05840		YrdB family protein	9.345932376	0.002147807
SMU_RS06125		IS982 family transposase	3.558950023	0.001112047
SMU_RS06130	mubP	Mutanobactin A biosynthesis phosphopantetheinyl transferase MubP	10.17901461	0.00029982
SMU_RS06135	mubJ	Mutanobactin A biosynthesis reductase MubJ	14.07860947	9.31925E-05
SMU_RS06140	mubI	Mutanobactin A biosynthesis transacylase MubI	16.9185711	6.01184E-05
SMU_RS06145	mubM	Mutanobactin A biosynthesis alpha/beta hydrolase MubM	19.02684342	5.528E-05
SMU_RS06150	mubZ	Mutanobactin A system MFS transporter MubZ	25.72647422	3.50961E-05
SMU_RS06245		Class I SAM-dependent methyltransferase	47.44451332	1.3553E-07
SMU_RS06485	lpdA	Dihydrolipoyl dehydrogenase	9.200686942	0.000451023
SMU_RS06490		ATP-dependent Clp protease ATP-binding subunit	5.616361678	0.000223691
SMU_RS06780	lacD	Tagatose-bisphosphate aldolase	8.619135052	0.005858252
SMU_RS08020	cas2	CRISPR-associated endonuclease Cas2	2.310182305	0.000173604
SMU_RS08025	cas1c	Type I-C CRISPR-associated endonuclease Cas1c	2.31468337	8.56152E-05
SMU_RS08030	cas4	CRISPR-associated protein Cas4	2.62406735	4.22101E-05
SMU_RS08035	cas7c	Type I-C CRISPR-associated protein Cas7/Csd2	2.551677073	1.09859E-05
SMU_RS08040	cas8c	Type I-C CRISPR-associated protein Cas8c/Csd1	2.404101888	1.46702E-05
SMU_RS08045	cas5c	Type I-C CRISPR-associated protein Cas5c	2.330583977	1.72895E-05
SMU_RS08050		CRISPR-associated helicase/endonuclease Cas3	2.004577825	6.18313E-05
SMU_RS08500	hdrR	LytTR family transcriptional regulator HdrR	2.427695352	0.00237442
SMU_RS09005	comGG	Competence type IV pilus minor pilin ComGG	8.980541676	1.92133E-06
SMU_RS09010	comGF	Competence type IV pilus minor pilin ComGF	6.476136433	2.85151E-06
SMU_RS09015	comGE	Competence type IV pilus minor pilin ComGE	6.308944389	6.02236E-06
SMU_RS09020	comGD	Competence type IV pilus minor pilin ComGD	6.11858782	5.36394E-07
SMU_RS09025	comGC	Competence type IV pilus major pilin ComGC	6.676134484	2.91046E-07
SMU_RS09030	comGB	Competence type IV pilus assembly protein ComGB	8.289440643	2.53468E-07
SMU_RS09035	comGA	Competence type IV pilus ATPase ComGA	3.892465777	1.78309E-05
SMU_RS09720		NAD-dependent succinate-semialdehyde dehydrogenase	2.011111598	0.004321426
SMU_RS10095		Hypothetical protein	4.734795738	0.042401635
SMU_RS10100		Hypothetical protein	3.466150245	0.028948456
SMU_RS10170		Hypothetical protein	5.448985825	0.000324982

^*a*^ Results were expressed as ratios of the *xre *mutant (X) over the wild type (W).

^*b*^ Results were expressed as ratios of the wild type (W) over the *xre* mutant (X).

The down-regulated genes include those for DNA repair and processing (i.e*.*, class I SAM-dependent methyltransferase and DNA-processing protein DprA), environmental stress tolerance response (LytTR family transcriptional regulator HdrR) (30), sugar metabolism (fructose/mannose-specific enzyme II), DNA uptake and competence development (ComGF/E/D/C/B/A) (31, 32), and biosynthesis and transport of mutanobactin (phosphopantetheinyl transferase MutP, reductase MutJ, transacylase MutI, alpha/beta hydrolase MutM, and MFS transporter MutZ). More than 18 genes, 5 up-regulated and 13 down-regulated, were found to encode hypothetical proteins with unknown functions. Of note, the *comG* genes and those for the mutanobactin synthesis and transport are arranged in apparent operons (SEED Viewer V2; https://rast.nmpdr.org/seedviewer.cgi?page=Home).

## DISCUSSION

The results presented here have shown that locus SMU.405c, encoding an XRE-like transcriptional regulator, plays an important role in *S. mutans* physiology, including oxidative stress tolerance response. Relative to the parent strain, the XRE-deficient mutant had a weakened ability to survive oxidative stressors, while it also had moderate increases in biofilm formation when growing in medium with sucrose. Luciferase reporter assays, *in vitro* transcription assays, RNA-seq, and RealTime-qPCR revealed that as a repressor and a positive regulator, the XRE-like regulator encoded in SMU.405c influences the expression of groups of genes, including *brpA,* a gene known to play a critical role in cell envelope biogenesis, and those involved in oxidative stress tolerance response and biofilm formation.

XRE response elements are among the most widespread regulatory elements in bacteria that control various aspects of bacterial physiology, including cell envelope biogenesis, oxidative stress tolerance, and biofilm formation (15, 18, 33). A transcription factor of the XRE family was recently found to function as a negative regulator of capsule polysaccharides synthesis in *Neisseria meningitidis* (18). Similarly, the results presented here suggest that the SMU.405c-encoded XRE-like regulator in *S. mutans* also plays a role in the regulation of the expression of BrpA, which is a member of the LytR-CpsA-Psr family proteins with a key role in cell wall-associated rhamnose-glucose polymer biogenesis, stress tolerance response, and biofilm formation (8, 10). It is noteworthy that while the results of RNA-seq did not show significant differences in *brpA* transcription in response to *xre* deletional mutation, which can be in part attributed to the low abundance of the *brpA* transcripts (34). RealTime-qPCR analysis of the ∆*xre* mutant total RNA did measure the *brpA* transcripts at a level >3.3-fold higher than the wild type, UA159 (*P* < 0.05), which is consistent with the results of luciferase reporter fusion assays.

The XRE family regulators are characterized by a highly variable C-terminal region and a conserved N-terminus with a helix-turn-helix DNA binding domain, which recognizes ACC(N0,7)GTT motifs in the promoter region of target genes (17, 33). A region designated as XRE-box in the promoter of *brpA* was also identified with homology to the binding motif of XRE-family proteins; and it is partly overlapping the Fnr-Box (13). The results of EMSA further suggest that XRE can indeed interact with the *brpA* promoter, modulating the expression of *brpA.* Consistently, both reporter fusion assays and RealTime-qPCR showed major increases in *brpA* expression in response to the deletional mutation of *xre*, indicative of XRE as a repressor in the regulation of *brpA* expression. This was further supported by the *in vitro* transcription assay that displayed the inhibitory effects of rXRE on *brpA* transcription; and the results also suggest that with the XRE repressor, *brpA* transcription likely fell off, resulting in the production of a short pre-mature transcript.

Interestingly, as indicated by the results of reporter gene fusion assays, the most significant derepression of *brpA* expression in response to *xre* deletion was measured when the reporter expression was under the direction of the promoter derivative with both the XRE-box and Fnr-box being deleted. It suggests that the XRE-like regulator may regulate *brpA* expression as a cofactor of a major regulatory complex, which is yet to be uncovered. The results showed reporter activity had no significant differences under the direction of the intact *brpA* promoter, while RealTime-qPCR did show significant increases in *brpA* transcripts in response to *xre* deficiency, also suggesting that *brpA* expression in *S. mutans* is regulated at both the transcriptional and translational levels. A major effort is being directed to elucidate how XRE and members of the regulatory complex govern the regulation of *brpA* expression.

*S. mutans* is known for its ability to survive and adapt to various environmental stressors; and multiple factors regulate oxidative stress tolerance response, including Rex, SpxA1, SloR, and PerR (22, 25, 35–37). Like what was recently reported in *Streptococcus suis* and others (15, 33), the results presented here showed that an XRE-like regulator (SMU.405c) in *S. mutans* also plays a role in oxidative stress tolerance response. Of the genes that were up- or down-regulated in response to the deletion of *xre*, as revealed by RNA-seq, several are known to play important roles in DNA repair and environmental stress tolerance response. These include up-regulation of excinuclease UvrC and down-regulation of class I SAM-dependent methyltransferase and DNA-processing protein DprA. UvrC is part of the excinuclease ABC complex, which is critical in excision repair and triggered in response to UV radiation and DNA damage (38). The SAM-dependent methyltransferase is known for its role in the methylation of DNA and proteins, a process that affects gene expression, survival, and other cellular functions. DNA processing protein DprA is crucial for natural transformation but also plays a significant role in DNA repair and bacterial virulence (39). HdrR is a LytR family transcriptional regulator with a role in environmental stress tolerance response (30). Mutanobactin is a lipopeptide that *S. mutans* uses to protect from reactive oxygen species; and deletional mutation of genes affecting mutanobactin biosynthesis has been shown to lead to increased sensitivity to oxidative stressors (40, 41). Therefore, these results suggest that oxidative stress tolerance response, including DNA processing and repair, is part of the XRE-regulon in *S. mutans*; and down-regulation of genes involved in oxidative stress tolerance, including DNA processing and repair, as revealed by RNA-seq, is likely part of the contributing factors to the weakened oxidative tolerance response observed with the *xre* mutant. The results presented here also suggest that while XRE is part of the regulatory complex governing the expression of brpA, its role in oxidative stress tolerance response is likely independent of BrpA. However, how XRE in *S. mutans* regulates its oxidative tolerance response awaits further investigation.

*S. mutans* possesses multiple pathways to adhere to and persist on the tooth surface, including the sucrose-dependent pathway involving the production and binding of adhesive glucose polymers from sucrose (2, 35). Central to *S. mutans* cariogenicity, the glucosyltransferases (GtfBC&D) catalyze the production of adhesive polymers that coat the tooth surface and microbial cells, while the glucan-binding proteins (GbpABCD) bind to the glucans, facilitating surface- and inter-cellular adherence and accumulation of multi-cellular clusters. The Gtf proteins can also bind to their own adhesive products. While not statistically significant, the ∆*xre* mutant did show enhanced biofilm formation when it was grown in the presence of sucrose, as compared to the parent strain, UA159. As revealed by RNA-seq and confirmed by RealTime-qPCR, up-transcription of GtfB and GbpC in response to *xre* deficiency is likely part of the contributing factors. A putative XRE-box was identified in the promoter region of *gbpC* but not *gtfB.* However, it awaits further investigation if the XRE-like regulator directly regulates the expression of *gbpC* and *gtfB,* or otherwise, their altered expressions were an indirect result in response to ∆*xre* mutation.

It is worth noting that biofilm formation by *S. mutans* is highly regulated, and environmental conditions, such as pH and oxidative stressors, are known to significantly influence biofilm formation and maturation. Considering the ∆*xre* mutant also had a weakened ability to adapt and survive oxidative stress, such a defect will likely have a major impact on its biofilm formation, especially in the presence of oxidative stressors. It awaits further investigation how the ∆*xre* mutant competes and persists when grown in the complex oral microbiota, of which some antagonistic species are known to produce hydrogen peroxide and other oxidative stressors in competition for nutrients and niches.

It is apparent that the SMU.405c product in *S. mutans* is a regulatory protein that modulates the function of multiple aspects of bacterial physiology. As a positive regulator, XRE-deficiency resulted in the reduction in expression of >78 genes, including those involved in DNA methylation and oxidative stress response; while it can also function as a repressor, regulating the expression of *brpA* and several other genes known to be critical to cell envelope biogenesis in *S. mutans* and its ability to form biofilms and cause caries. Of the altered genes, 10 encode hypothetical proteins whose functions are currently unknown. Of note, among the down-regulated genes were the *comGF/E/D/C/B/A* (also *comYF/E/D/C/B/A*) cluster, likely co-transcribed as an operon, which encodes proteins involved in DNA uptake and competence development. To investigate if XRE-deficiency imposed any major defects on genetic competence, the deficient mutant, along with the parent strain, UA159, was examined using a transformation assay with and without inclusion of competence-stimulating peptides. However, the results showed no major differences between the ∆*xre* mutant and its parent strain, indicative of no major impact of XRE-deficiency in DNA transport and genetic competence development.

XRE-type transcriptional regulators are widely recognized for their ability to be regulated by both ligand binding and post-translational mechanisms, allowing them to control complex bacterial behaviors in response to environmental changes. XRE-type regulators are featured with a conserved N-terminus with Helix-Turn-Helix DNA-binding domain and a diverse C-terminus which is crucial for dimerization. Additionally, the C-terminal domains are also involved in sensing environmental signals, such as oxidative stressors and metabolites; and some possess a C-terminus with a “cupin” fold, called XRE-cupin, which typically senses metabolites that are part of the pathway they regulate (42). For example, PsdR in *Pseudomonas aeruginosa* is involved in dipeptide metabolism, and PauR senses diamines, such as putrescine and cadaverine (43). Some XRE regulators detect specific amino acids, such as D-methionine, which has been identified as a ligand bound to an XRE-cupin regulator from *Vibrio cholerae*. XRE regulators can sense various stressors, including oxidative stressors and DNA damage. The XRE-like regulator encoded by SMU.405 does not seem to have a “cupin” fold, although what signals and how they trigger the XRE-mediated regulation in *S. mutans* await further investigation. In addition, there is evidence suggesting post-translational control of XRE regulation via protein-protein interactions. It is also worth noting that oral bacteria face constant challenges of oxidative stressors, such as hydrogen peroxide and that while hydrogen peroxide is commonly used in studies of bacterial oxidative stress tolerance response, the concentration used for this study does not necessarily reflect the physiological conditions in the oral cavity.

In summary, functional genomics, along with molecular and transcriptomic analysis, has generated evidence for the first time that the XRE-like transcription factor encoded in SMU.405c plays an important role in the regulation of various aspects of *S. mutans’* physiology, including oxidative stress tolerance response. However, the exact role and underlying mechanisms of the XRE (SMU.405c)-mediated regulation in *S. mutans* pathophysiology await further studies under more relevant conditions.

## SUPPLEMENTAL MATERIAL

Supplemental material spectrum.00116-26-s0001.pdf Fig. S1 to S5; Table S1.

ASM does not own the copyrights to Supplemental Material that may be linked to, or accessed through, an article. The authors have granted ASM a non-exclusive, world-wide license to publish the Supplemental Material files. Please contact the corresponding author directly for reuse.
