# Supplementary material for: An XRE-type regulator in Streptococcus mutans plays an important role in brpA expression and oxidative stress tolerance response
Source: Microbiol Spectr. 2026 Jul 21;14(9):e00116-26. doi: 10.1128/spectrum.00116-26 (PMC13532258; doi:10.1128/spectrum.00116-26)
Supplement: Supplementary file 1 [file spectrum.00116-26-s0001.pdf]

**Table S1. Primers used in this study**

| Name | Oligonucleotide sequence (5'-3') | Application |
|---|---|---|
| XRE55 | atctgttgagaaactgcatgctcttc | XRE allelic exchange mutant |
| XRE53Xb | atcgaacatctagataagacaac | XRE allelic exchange mutant |
| XRE35Xb | cttctagagttagatgggattgttgg | XRE allelic exchange mutant |
| XRE35St | cttcttgagctcgatgggattgttgg | XRE allelic exchange mutant |
| XRE33 | tcaagagcaaggacacttggttatc | XRE allelic exchange mutant |
| XREe3Sal | tgttgtgtcgacattacaatcaccttc | XRE expression |
| XREe5RI | atgaattcattggagaaaaactcaaaacagc | XRE expression |
| XREc5St | acgagctcaatcatgaaatggaaatc | XRE mutant complementation |
| XREc3Kp | cattatttggtacctcatcattacaatc | XRE mutant complementation |
| brpA fw | cgtgaggtcatcagcaaggtc | RealTime-qPCR |
| brpA rv | cgctgtaccccaaaagtttagg | RealTime-qPCR |
| 16S rRNA fw | cacaccgcccgtcacacc | RealTime-qPCR |
| 16S rRNA rv | cagccgcaccttccgatacg | RealTime-qPCR |
| gtfB fw | agcaatgcagccatctacaaat | RealTime-qPCR |
| gtfB rv | acgaactttgccgttattgtca | RealTime-qPCR |
| gbpC fw | atacacaagcatcagaac | RealTime-qPCR |
| gbpC rv | attagcattagccttagc | RealTime-qPCR |

Note: Sequences underlined are restriction sites engineered for cloning.

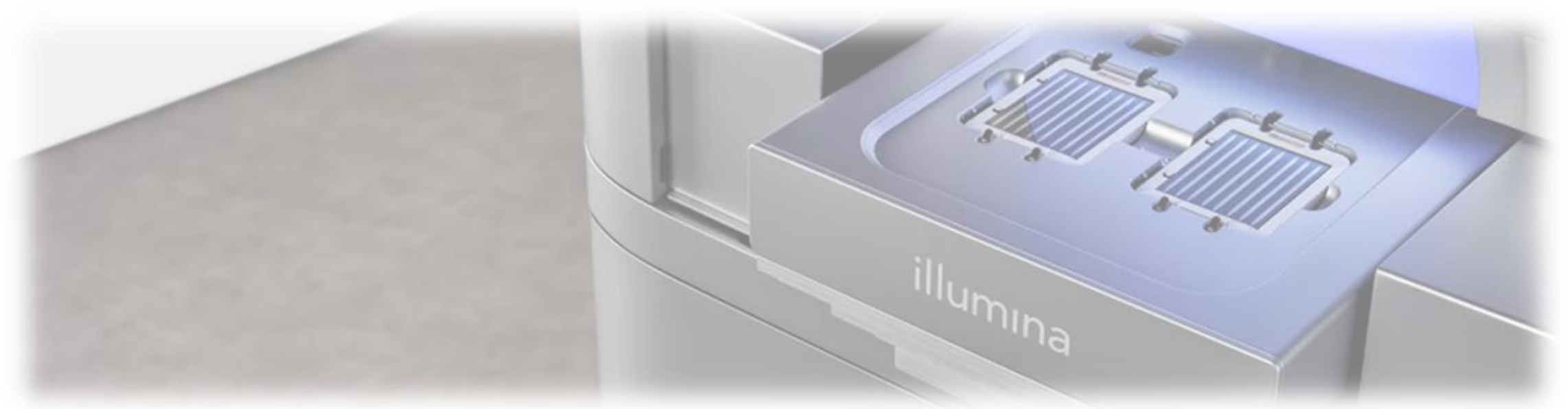


SEQCENTER

## RNA Sequencing Methods

RNA extractions, if applicable, follow the methods reported on Page 2.

Samples were DNAse treated with Invitrogen DNAse (RNAse free). Library preparation was performed using Illumina's Stranded Total RNA Prep Ligation with Ribo-Zero Plus kit and 10bp unique dual indices (UDI). (If arrangements were made in advance to use custom probes, they were used in conjunction with the standard modules.)

Sequencing was done on a NovaSeq X Plus, producing paired end 150bp reads. Demultiplexing, quality control, and adapter trimming was performed with bcl-convert (v4.2.4)[1]. Sequencing statistics are included in the '*RNA Sequencing Stats.xlsx*' file.

No additional trimming or filtering was performed. If you received analysis on your RNA data post-sequencing you will have received a separate text document with your analysis files describing the methods and version numbers used for your samples.

What is an md5sum?

The md5sum functions as a file's compact digital fingerprint. md5sums are used to verify the integrity of files between two servers. If you calculate the md5sum of the file on your server, it should match that of the file listed in the '*DNA Sequencing Stats.xlsx*' file. If it does not match, the file was corrupted either being uploaded or downloaded to box, please let SeqCenter know if this is the case.

Calculating the md5sum:

- Windows users, please see this tutorial.
- MacOS users, please see this tutorial.
- Linux users, please see this tutorial.

91 43rd Street
Suite 250
Pittsburgh, PA 15201
878-227-4915

[1] bcl-convert: A proprietary Illumina software for the conversion of bcl files to basecalls.

## Basic RNA-Seq Analysis (Prokaryote)

Read mapping was performed with HISAT2[1]. Read quantification was performed using Subread's featureCounts[2] functionality. HISAT2 output files and BAM alignment files can be found in the respective sample folders.

Summary mapping statistics can be found in the 'alignment.tsv' file. Summaries of the raw, quantified CDS counts are reported in the 'hisat2_counts.tsv' file. Gene information extracted from the provided annotation file is reported in the 'gene2description.tsv' file.

| Tool | Version | Parameters |
|---|---|---|
| hisat2 | 2.2.1 | default parameters + '--very-sensitive' |
| subread | 2.0.6 | featureCounts; default parameters + '-Q 20' |

[1] Kim, D., Paggi, J.M., Park, C. et al. Graph-based genome alignment and genotyping with HISAT2 and HISAT-genotype. Nat Biotechnol 37, 907–915 (2019).
[2] Liao Y, Smyth GK and Shi W. featureCounts: an efficient general-purpose program for assigning sequence reads to genomic features. Bioinformatics, 30(7):923-30, 2014

91 43rd Street
Suite 250
Pittsburgh, PA 15201
878-227-4915

## Intermediate RNA Analysis with Replicates (Prokaryotic)

Quality control and adapter trimming was performed with bcl-convert[1]. Read mapping was performed with HISAT2[2]. Read quantification was performed using Subread's featureCounts[3] functionality. Mapping statistics can be found in the '*alignment.tsv*' file. Raw, quantified counts are available in the "*hisat2_counts*.tsv" file. Read counts loaded into R[4] were normalized using edgeR's[5] Trimmed Mean of M values (TMM) algorithm. Subsequent values were then converted to counts per million (CPM). Differential expression analysis was performed using edgeR's glmQLFTest. The file "*All Quantified Genes.tsv*" contains the results of the glmQLFTest for all genes in addition to the normalized counts per million. The file "*Differentially Expressed Genes.tsv*" is a subset of the aforementioned file with |logFC| > 1 and p < .05 (log fold change is base-2). The differentially expressed gene's normalized counts per million were then used to create a heatmap, "*Differentially Expressed Genes Heatmap.tiff*". The PCA, "*Sample PCA.pdf*" is based on the global expression of all genes in the "*All Quantified Genes.tsv*" file. No pathway analysis could be performed for your selected reference.

| Tool | Version | Parameters |
|---|---|---|
| bcl-convert | 4.2.4 | default parameters |
| hisat2 | 2.2.0 | default parameters + '--very-sensitive' |
| featureCounts | 2.0.1 | default parameters + '-Q 20' |
| R | 4.0.2 | default parameters |
| edgeR | 1.14.5 | default parameters |

[1] bcl-convert: A proprietary Illumina software for the conversion of bcl files to basecalls.
[2] Kim, D., Paggi, J.M., Park, C. et al. Graph-based genome alignment and genotyping with HISAT2 and HISAT-genotype. Nat Biotechnol 37, 907–915 (2019).
[3] Liao Y, Smyth GK and Shi W. featureCounts: an efficient general-purpose program for assigning sequence reads to genomic features. Bioinformatics, 30(7):923-30, 2014
[4] R Core Team (2020). R: A language and environment for statistical computing. R Foundation for Statistical Computing, Vienna, Austria. https://www.R-project.org/
[5] Robinson MD, McCarthy DJ and Smyth GK (2010). edgeR: a Bioconductor package for differential expression analysis of digital gene expression data. Bioinformatics 26, 139-140

91 43rd Street
Suite 250
Pittsburgh, PA 15201
878-227-4915

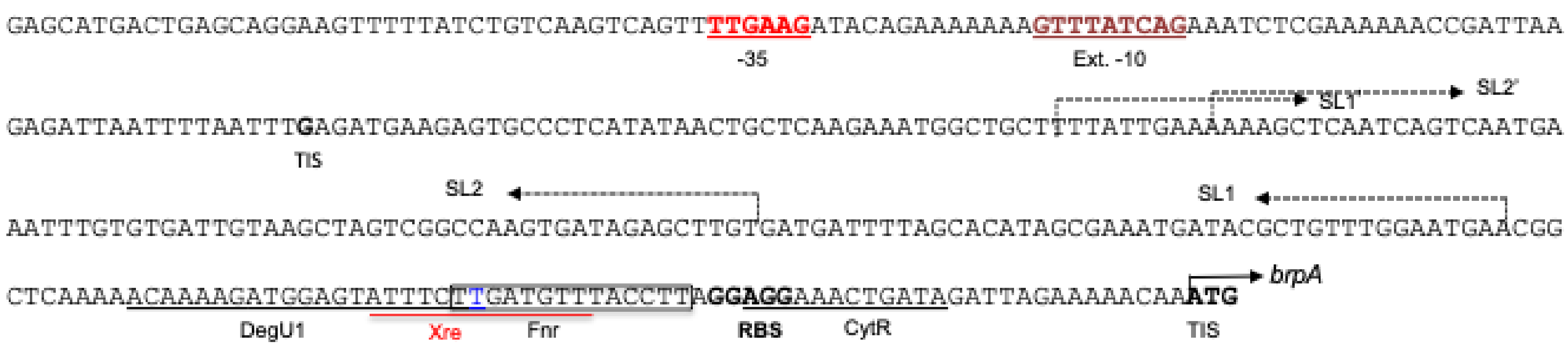


**Figure S1**. Relevant nucleotide sequences and features of the short *brpA* promoter. Underlined in dark brown and red faces are the predicted -10 and -35 regions. The transcription initiation site (TIS), the putative Shine-Dalgarno sequence (RBS), and translational initiation site ATG (TIS) are indicated in bold face. Potential binding sites for transcriptional regulators CytR, Fnr, Xre, and DegU are boxed or underlined, as indicated. Two inverted repeats, SL1-2, are illustrated by dashed arrows.

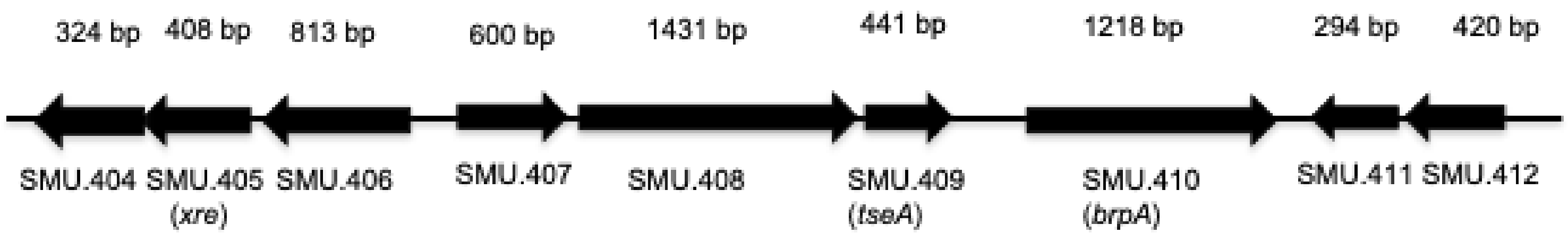


**Figure S2.** Schematic diagram of the *xre*-flanking region in the genome of *S. mutans* UA159. The arrows indicate the orientation of the respective genes with the locus number (gene names) underneath and the numbers above indicating the sizes of the different coding regions.

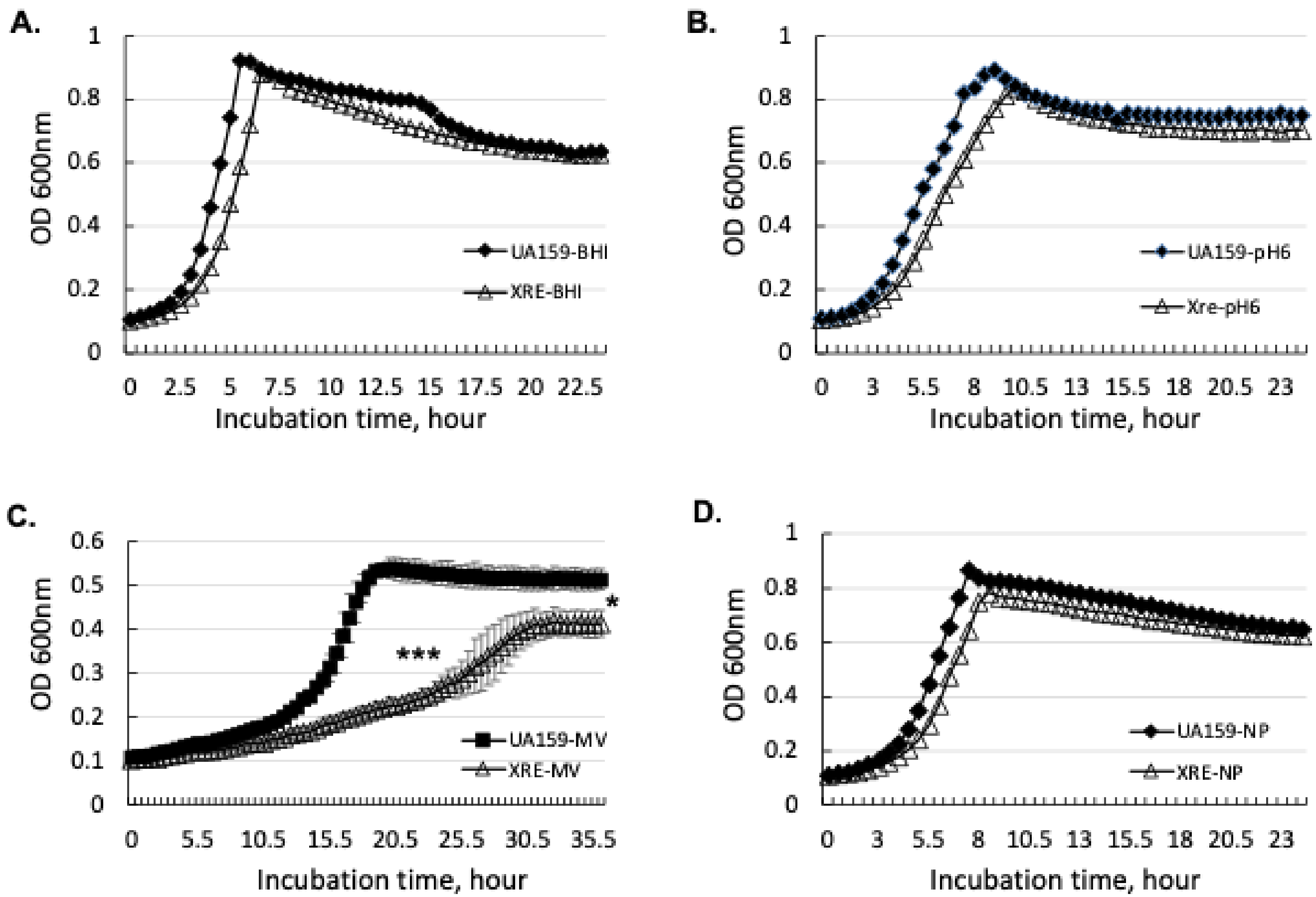


**Figure S3**. Growth characterization of the Δ*xre* mutant using a Bioscreen C. *S. mutans* UA159 and its Δ*xre* mutant (XRE) were grown in regular BHI broth (BHI, **A**), BHI with pH adjusted to 6.0 (pH6, **B**); BHI containing methyl viologen at 25 mM (MV, **C**), and BHI with inclusion of sodium nitroprusside (NP, **D**). Data presented here are representatives of three independent experiments with the Δ*xre* mutant showing significant reduction in growth rate (***, $P<0.001$) and optical density (*, $P<0.05$) when growing in the presence of MV, as compared to its parent strain, UA159. No significant differences were measured when growing in BHI, BHI-pH 6 and BHI-NP.

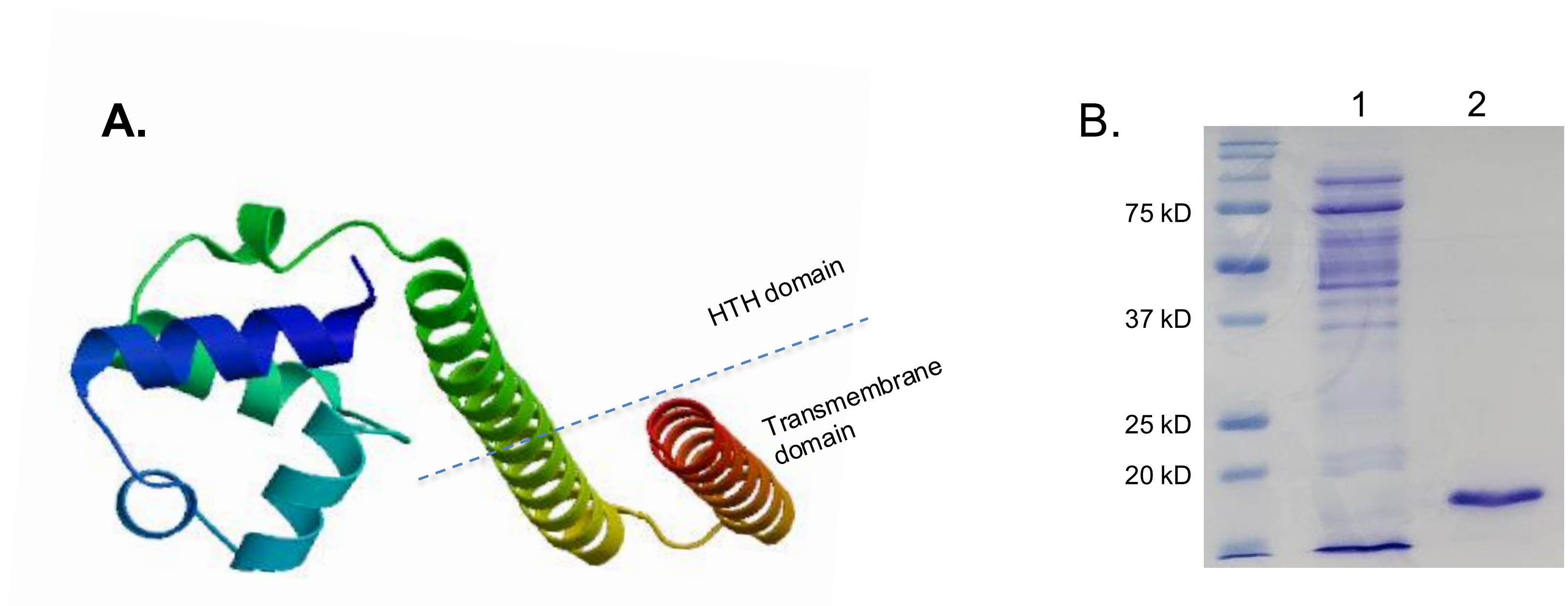


**Figure S4.** Protein domain prediction (**A**) and purification (**B**) of XRE. **A.** The XRE-like protein was predicted via SWISS MODEL to possess a XRE HTH-domain from aa 7 to 61 with four $\alpha$-helices and a transmembrane domain from aa 88-106 and aa 112-132 (http://swissmodel.expasy.org/interactive/mUrbup/models/). **B.** rXRE was expressed in *E. coli* M15 as a his-tagged protein via pQE30 and purified via native condition. Graph shows 15% SDS-PAGE analysis of the purified protein (lane 1, flow through; lane 2, elution fraction 3).

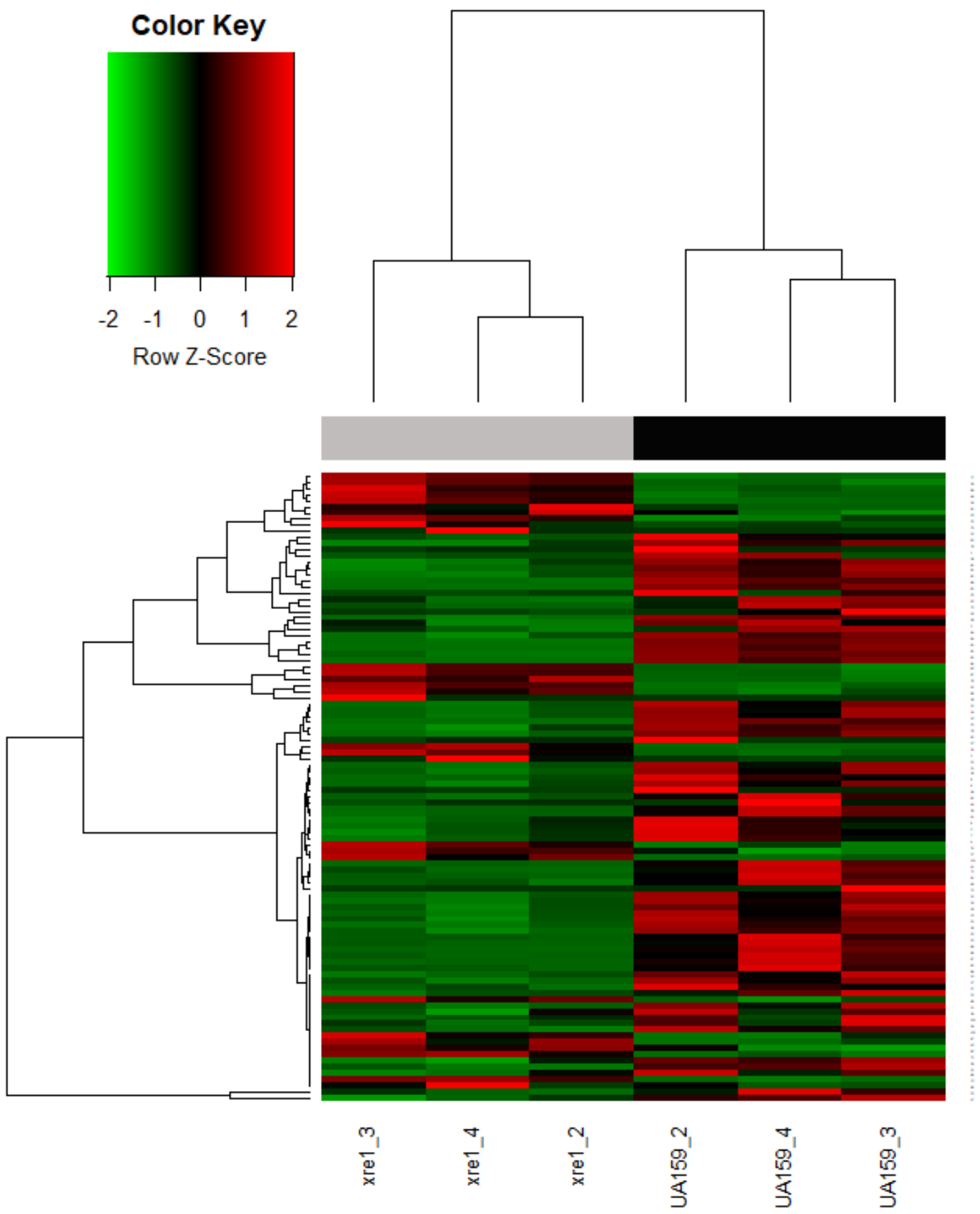


**Figure S5**. Differentially Expressed Genes Heatmap. It was created using the differentially expressed gene's normalized counts per million.
